# The Bristol Royal Infirmary

**Published:** 1906-02-24

**Authors:** 


### HOSPITAL ADMINISTRATION.

### CONSTRUCTION AND ECONOMICS.

### THE BRISTOL ROYAL INFIRMARY.

#### WHY NOT AN UP-TO-DATE MODERN HOSPITAL?

Chiefly owing to the energy and devotion of Sir George White, the Chairman, the sum of £50,000 is now practically in hand to enable the reconstruction of the old buildings to be proceeded with. The present buildings are in many respects out of date, several of the wards leaving much to be desired. The operation theatres, too, though beautifully kept, do not fulfil the conditions imposed upon those responsible for the erection of a modern hospital in the present day. There is no casualty department worthy of the name, and, taken as a whole, the buildings are not worthy of the importance and requirements of the ancient city of Bristol. In such circumstances it is a serious matter to take in hand the reconstruction of old buildings on a limited site, such as that which has to be dealt with in this case. We hope that Sir George White and his advisers will take infinite care to secure, that not one penny of the £50,000 shall be spent, until a complete scheme has been prepared, which shall provide for the rebuilding of the whole hospital so as to make it as perfect as possible.

It is unfortunately possible, as experience proves, to spend even a larger sum than £50,000 on the reconstruction of old buildings without materially improving the accommodation or rendering it possible to hope that it will be adequate for the purposes of the hospital for any lengthy period of time. The governors of the Bristol Royal Infirmary have a great opportunity, by the exercise of patience and the exhibition of brains, to secure a perfected scheme which shall deal with every part of the hospital, and, by utilising all the site, scattered though it is, which the governors can command, to employ the £50,000 to secure a thoroughly modern hospital. The Bristol Royal Infirmary is at the parting of the ways. If great wisdom and ability are shown, the £50,000 may be the means of ultimately providing a thoroughly modern hospital, replete in every department. It may, on the other hand, be easily expended on patchwork and tinker, with the result, that the reputation and credit of the Bristol Royal Infirmary must be so damaged as to ultimately destroy the influence and most of the usefulness of this ancient foundation.

Sir George White has shown commendable energy in raising this £50,000, and we hope that he will set his face against the expenditure of any portion of this large sum until he has evolved, with the aid of the medical staff, and such expert advice as may be available, a scheme of reconstruction which, from its inherent attractiveness and ability, will win for it the support of all whose opinion is best worth having. The governors of the Birmingham General Hospital had very nearly the same problem to solve which is now presented to the governors of the Bristol Royal Infirmary. Had they not shown commendable courage and public spirit by determining to remove the hospital and rebuild it upon a more suitable and adequate site, they could never have commanded the success which has ultimately crowned their efforts. Few if any experts of large experience can visit the Royal Infirmary at Bristol and make a careful inspection of the buildings, and of the scattered site which the governors control, without coming to the conclusion, that it would be far better not to spend any of the £50,000 at the moment, than to adopt merely palliative measures, whereby the whole sum will be eaten up and the infirmary—from a hygienic and modern hospital standpoint—left probably pretty much where it has been, so far as buildings are concerned, for the last 20 years or more. We are confident that if Sir George White has the courage to stiffen his back and refuse to sanction any merely palliative measures, whilst he devotes his energies to the preparation of an exhaustive and adequate scheme of reconstruction, which will embrace every department of the institution, and ultimately make it the most complete institution of the kind in the West of England, he will eventually secure, as he deserves, complete success.

There must be a great number of people of wealth and position in the district who would be willing to undertake to provide money for the erection of a block of buildings to commemorate the name of some beloved member of their family, or to perpetuate his work in the district. A complete scheme might be so subdivided as to buildings as to give ample opportunities for such generous donors to undertake particular sections of the work for memorial purposes. This has been done with increasing success in the United States, and a few of the larger hospitals have found the plan very successful in this country in recent years. It is a good thing that Sir George White and his friends should have raised £50,000; but if the utmost benefit is to be derived from this money, it can only be done by following the lines which we have ventured to indicate in this article. Having regard to the existing resources of the governors of the infirmary, the money already collected, and to the actual cost of carrying through an adequate scheme which would provide Bristol with a modern and up-to-date hospital, we have some confidence that Sir George White may see his way to refuse to permit patchwork additions and alterations. In such an event he will set his mind to the task of rebuilding the whole of the wards, and of utilising such portions of the old infirmary as may be suitable to the purposes of an administration building. It would be wise for a few members of the Board and medical staff to visit and inspect the Northampton County Hospital, and to see for themselves how much has been accomplished there by an expenditure of less than £35,000.

